# McKinsey and the ‘Tripartite Monster’: The Role of Management Consultants in the 1974 NHS Reorganisation

**DOI:** 10.1017/mdh.2019.41

**Published:** 2019-10

**Authors:** Philip Begley, Sally Sheard

**Affiliations:** Department of Public Health and Policy, University of Liverpool, Whelan Building, Liverpool, L69 3GB, UK

**Keywords:** National Health Service, Management consultancy, Policymaking, Reform, Reorganisation, Expertise

## Abstract

The first major reorganisation of the National Health Service took place in 1974, twenty-six years after the service had been established. It has long been perceived as a failure. This article draws on archival records and a witness seminar held in November 2016 to provide a more nuanced assessment of the 1974 reorganisation and understand more fully why it took the form that it did. In particular it identifies the reorganisation as an important moment in the ongoing story of management consultants engaging with health policymakers, and explores the role of McKinsey and Co. in detail for the first time. Key explanatory factors for their involvement are identified, including the perceived lack of expertise and manpower inside the civil service and the NHS, and perceptions of their impact and effectiveness are discussed. Many debates about the use of management consultants today were directly foreshadowed during the early 1970s. Alongside this, the role of other groups of policy actors, including civil servants, politicians and medical professionals, are established and the extent to which British health policymakers have had to work within existing cultural, political, legislative and practical constraints when trying to initiate change is demonstrated. The fact that many of the ‘mistakes’ that were made have been repeated in the course of subsequent reforms, speaks to the poor institutional memory of Whitehall, and the Department of Health and Social Care in particular. In the run up to 1974 management consultants could make only a limited contribution to an imperfect compromise.

## 1 Introduction

Since its creation in 1948 the National Health Service (NHS) has been reformed many times. The basic principles that underpin it may remain the same, but its structures and processes have changed significantly. As Nicholas Timmins has noted, ‘“organisation, re-organisation and re-disorganisation” might well be dubbed the NHS disease’.^1^ This article examines the first major reorganisation of the service in 1974. In particular it identifies the reorganisation as an important moment in the ongoing story of management consultants engaging with health policymakers. The use of management consultants has increased significantly over time. They have since become culturally embedded in the NHS and in Whitehall; able to replace or supersede internal expertise, make connections with policymakers (including ministers and their special advisers), and take advantage of the opportunities presented by managerial and market-oriented reform and the development of a normative culture in which external consultants have a significant role. This rise has had implications for the quality, cost and accountability of advice, as well as wider issues around health policy formation and public governance in Britain. It has been underpinned by the perception that management consultants are able to understand complex institutional change and offer worldwide policy learning across organisations and between the public and private sectors.^2^ There is also a tangible sense in which the use of management consultants serves to add prestige and legitimacy to official decisions or can help to redistribute attention and responsibility when addressing difficult questions.^3^

In 2014 it was widely reported that spending on consultants had reached £640m per year.^4^ That figure has since fallen as part of wider spending constraints, but consultants remain important players. For example, they have come to have a key role in the development of Sustainability and Transformation Partnerships.^5^ Yet the 1974 reorganisation appears to be the first time that management consultants were engaged at such a high level on a project of such importance in the health field. Strikingly, the work of McKinsey & Co., the management consultancy firm employed by the Department of Health and Social Security (DHSS) during the preparations for the 1974 reorganisation, has almost become synonymous with the reforms. Even Kenneth Clarke, who became Secretary of State for Health in 1988, has referred to the ‘stupid McKinsey report’ and the ‘McKinsey reforms’.^6^ However, it has not always been clear what McKinsey’s contribution actually was.

In historiographical terms, the reorganisation and its many political and organisational developments, have long been well covered in histories of the NHS by Charles Webster, Geoffrey Rivett, Rudolf Klein and others.^7^ The 1973 National Health Service Reorganisation Act replaced the original tripartite structure, which had separated primary care, secondary care and local health services, with a more integrated model that brought services together under new regional and area health authorities and district management teams.^8^ Meaningful progress was achieved, but integration was not complete and significant gaps remained, while new problems were created.

Important contemporary accounts were published by Ruth Levitt and Ronald Brown.^9^ Levitt documented the changes in great detail in 1976, including the myriad roles and responsibilities of the new layers of administration, and the implications for different medical services, NHS staff and the public. Tellingly, she also identified a number of ‘unresolved problems’ around planning and collaboration.^10^ By 1978 Brown was able to provide further details on the ‘machinery of change’ and had conducted a field study in Humberside, which highlighted delays in filling new posts and practical difficulties around establishing the necessary professional machinery. According to Brown, ‘The central architects of reorganisation gave a great deal of thought to new structures and processes as a vehicle for change, but not nearly enough to people, inducements and tools’.^11^ Subsequent studies, such as that by Jane Lewis, have also demonstrated a resulting decline in ‘community medicine’ and a negative impact on public health, while Stephen Harrison documented the problematic nature of ‘consensus management’, a central element of the reforms that is discussed in more detail in below.^12^

As such, the 1974 reorganisation has been widely remembered as a failure. A witness seminar held in 2016 largely confirmed this view.^13^ Klein’s observation that ‘the attempt to please everyone satisfied no one’ also speaks to the well-established perception in the literature that the reorganisation was a product of compromise between different interest groups – civil servants, medical professionals and NHS staff.^14^ However, despite the volume of material on the 1974 reorganisation, the involvement of McKinsey has only ever been discussed relatively briefly. How do we square this with the fact that more widely the firm have come to be so closely associated with the reforms and their failure? There has not yet been a detailed account of McKinsey’s role. This article examines it in depth and demonstrates that this sense of synonymity is not necessarily well founded. Nonetheless, it also shows that although McKinsey were not yet performing the wide ranging and more controversial role that management consultants would take on in future, many debates about the use of external consultants today – the lack of expertise inside the NHS, the apparent lack of accountability and responsibility, cost and pressure on resources, dependency and the quality of consultancy advice – were directly foreshadowed during the early 1970s. Such concerns were present much earlier than has so far been appreciated.

Alongside this, the article provides a new and more nuanced account of why the reorganisation took the form that it did. It achieves this by analysing the context of the period and the institutional cultures inherent to health policymaking at the time, examining the different motives of those involved and the forces pulling them in different directions. It draws on underused archival records and the 2016 witness seminar, which – while several important figures had sadly passed away during the intervening period – brought together twelve invited participants – medical professionals, civil servants, government ministers, NHS administrators and former McKinsey consultants – who were directly involved in the reorganisation or experienced it first-hand. It was chaired by Nicholas Timmins, a social policy journalist and historian, and followed a semi-structured agenda over four hours. Three McKinsey representatives participated: Robert Maxwell, Christopher Stewart-Smith and James Lee. Methodologically, the witness seminar allowed individual memories to be shared and contested in order to construct a fresh analysis of the 1974 reorganisation. The witnesses discussed the origins of the reforms, their introduction, effects and subsequent legacy. In the audience there were a further twenty individuals who contributed their views during open discussion. Additional oral history interviews were also conducted, including with McKinsey consultants Henry Strage and John Banham.

As will be discussed, the reforms were influenced by broad conceptions of post-war planning and rationalisation and an openness to what were perceived to be outside expert voices, such as management consultants.^15^ However, this was often underpinned by practical rather than philosophical considerations. McKinsey were only one of several expert groups feeding into the policymaking process. The importance of different types of policy actors to the 1974 NHS reorganisation has not yet been fully appreciated. This article underlines the extent to which health policymakers in Britain have often had to work within cultural, political, legislative and practical constraints when trying to initiate change. While working alongside civil servants and representatives of the health service and the medical professions, for example, management consultants could make only a limited contribution to an ultimately imperfect compromise.

## 2 Reform on the Agenda

The tripartite structure of the NHS established in 1948 was itself a product of compromise. While the inter-war years had seen a growing recognition on the part of health policymakers that greater efficiency, better treatment and wider coverage required a move to a comprehensive national health service, it took much longer to convince the medical profession, who had long been hostile to any new service being under local government control lest it should undermine clinical independence. In Webster’s words, ‘Intractable medico-realities stood in the path of enlightened planning goals’.^16^ After the groundwork laid by the wartime coalition government, particularly the 1942 Beveridge Report, Aneurin Bevan, Labour Minister of Health from 1945, succeeded in pushing through a significant degree of integration and effective nationalisation, at least with regard to the hospital service. The nettle of bringing general practitioners (GPs) into the NHS as salaried doctors and creating a fully integrated system was not grasped. Bevan compromised with senior doctors and ‘stuffed their mouths with gold’ to secure a settlement.^17^ GPs retained their position as independent contractors and were organised instead under separate Executive Councils. Public health remained the responsibility of local government. Though a significant step forward, the tripartite structure introduced in 1948 left plenty of scope for disorganisation.

The lack of communication produced by the separation of hospital and community services was particularly felt in services for the elderly and expectant mothers.^18^ Nonetheless, assessments of the early years of the NHS have usually been positive, not least in comparison to the fragmented service it replaced. According to Webster:

It was a remarkable improvement on the chaotic market arrangements that had existed before the Second World War. Despite evident shortcomings in the new system and the handicap of an environment of severe austerity, the regional hospital service succeeded in keeping up with the demands of modernisation and record of achievement, despite all of its idiosyncrasies.^19^

The most significant problem appeared to be that the early NHS might be the victim of its own success. The demand for new treatments and services had been underestimated, with the disparity between public take-up and official expectations made worse by the necessarily tight funding constraints of the post-war period. However, the 1956 Guillebaud Report, commissioned to examine the long-term financial viability of the NHS, concluded that the demographic and resulting financial pressure could largely be alleviated by better funding and greater management oversight in the running of the service.^20^ It did not yet prescribe a fundamental reorganisation. Debates about the future of the NHS continued to bubble under the surface.

By the mid-1960s pressure began to build for reform. The 1962 Porritt Report, produced by representatives of the leading medical organisations, called for a more unified service.^21^ Arthur Porritt himself was president of the British Medical Association and the Royal College of Surgeons. The report’s recommendations demonstrated the extent to which the settlement reached in 1948 was seen as imperfect and ultimately dispensable by senior doctors. As the Porritt Report confirmed, the tripartite foundations of the NHS were not set in stone:

The most vital need is to unify and integrate, in the widest sense, all aspects of medicine in order to achieve the highest standard of medical care and to avoid the sense of isolation and frustration to which we have drawn attention. Co-ordination and understanding between the profession and the administration is essential. No service can thrive if there is not a genuine partnership between these two vital elements. Our review has convinced us that a major fault in the National Health Service is its present tripartite administration, for this has led to difficulties in co-ordination and co-operation.^22^

The best available answer to these problems, while retaining the elements of the NHS that were popular and worked well, was, it was argued, integration of all the medical and ancillary services in a geographical area under a series of autonomous Area Health Boards.

On the ground there was also growing recognition of the need for reform. Dr John Marks, then a GP in Boreham Wood and chairman of Hertfordshire Executive Council, described at the witness seminar how he saw integration as being ‘desperately’ needed:

We had a tripartite system and one third did not know what the other two thirds were doing and vice versa …I did not have a clue what was going on in the hospitals, and as for the public health, they were a different world.^23^

The development of new towns particularly exposed the deficiencies of the existing structure. Dr Geoffrey Rivett, later a senior official at the Department of Health, but a GP in Bletchley in the late 1960s, was impressed by the local Medical Officer of Health (MOH), who brought together a group of local GPs, health authority and hospital representatives to plan medical services for Milton Keynes.^24^ Progressive MOHs were able to find ways to operate within the system to the benefit of their local community, for example moving local authority staff such as health visitors into GP surgeries,^25^ but not every area could boast individual leaders who found ways to make things work.

Recognition of the need for change was also seeping into the political sphere. By the late 1960s David Owen, a Labour MP, qualified doctor and future Minister of Health, had begun to strongly criticise the ‘tripartite monster’ that was holding back patient care.^26^ The slow development of community care facilities and relative lack of improvement in psychiatric hospitals were particular concerns, particularly after scandals such as that at the Ely Hospital in Cardiff.^27^

There were also moves to try and ‘modernise’ the NHS in a wider sense, which culminated in a series of reports and initiatives during 1967. The Salmon Report sought to improve the way hospitals were run by giving more power to nurses and encouraging senior nurses to become managers, and the first Cogwheel Report aimed to improve the organisation of doctors in hospitals by introducing specialist groups.^28^ Both recognised the difficulties thrown up by the tripartite system.

In 1968 Minister of Health Kenneth Robinson announced that the structure of the NHS would finally be studied alongside an expected reorganisation of local government. The 1969 Redcliffe–Maud Report formed a significant part of the wider post-war discourse around planning, and with local government reform came a natural expectation of complementary reform to the NHS.^29^ A long-term study group was established that helped to produce the first reorganisation Green Paper.^30^ It proposed replacing Hospital Management Committees and Regional Hospital Boards with between forty and fifty Area Boards, which would unify and administer all health services in England and Wales. A new focus on management was at the heart of the proposals, with little emphasis placed on the involvement of medical professionals in decision making.^31^ The ‘mood music’ of integration was clear.^32^ But objections were quickly raised by senior doctors, who once again feared being brought under the control of local government. Robinson’s plans were subsequently abandoned in a second Green Paper published in 1970 by Richard Crossman, who had become Secretary of State at the new DHSS in 1968.^33^ Ninety Area Health Authorities (AHAs) were now proposed, which would match the boundaries of the ninety new local authorities; a process known as co-terminosity. The AHAs would be the main centres of administration, supplemented by fourteen Regional Councils with a planning and advisory role, but which sat outside the main chain of command. There would also be 200 District Committees, which would bring in an element of local representation. The earlier managerial focus was now superseded by acceptance of the need for wider representation of clinicians on the boards of the new authorities.^34^

However, Crossman did not have a chance to see the reforms through. After Labour lost office in June 1970, Sir Keith Joseph, the incoming Secretary of State, took on the reforms. Changes were made in areas where the Conservatives disagreed with Labour. Despite such differences, however, a sufficient modicum of consensus existed between the two main political parties, leading representatives of the medical profession and NHS staff. As local government reform continued apace, a 1971 consultative document retained the idea of moving local authority services into new area authorities with coterminous boundaries with local government, but a stronger, integrated, regional tier was also introduced.^35^ This, it was hoped, would create a buffer between the powerful new AHAs, which would have an overview of all their local service needs, and those further up the chain (politicians and civil servants in DHSS), who would likely have different priorities.^36^ Teaching hospitals and public health services would also now be under the control of the AHAs, but local government retained its environmental health role. GPs were to remain separate as independent contractors under new Family Practitioner Committees. Once again, that particular ‘integration’ nettle was left un-grasped.

## 3 The Management Study

Ahead of the publication of a White Paper that would set out the detailed case for reorganisation, DHSS initiated a ‘management study’ to make recommendations for the internal organisation and management processes of the new regional, area and district levels.^37^ A further working party looked at collaboration between the health authorities and local authorities.^38^ Civil servants were well aware of the size of the administrative task they faced and called in reinforcements.

A great deal of thought went into the composition of the steering committee that oversaw the management study. Members were drawn from across the different civil service divisions in DHSS, and, although nominally invited by the Secretary of State in an individual capacity, representatives of different parts of the NHS were handpicked. While the final administrative decisions were to be taken by civil servants, bringing others in worked on two levels:

NHS Authorities and officers must be closely associated with the arrangements, with the double object of making sure that the recommendations make NHS sense and at the same time of giving the DHSS’ final decisions a greater authority than they otherwise would have.^39^

William Naylor of Sheffield Regional Hospital Board (RHB) was earmarked early on for the RHB Secretary spot because of his managerial approach and interest in reform, which was known to officials.^40^ Elizabeth Few, Chief Nursing Officer (CNO) in Buckinghamshire, was recommended for the local authority CNO position.^41^ P.M. Cooke, Group Secretary of Ipswich Group Hospital Management Committee, was recommended for his position.^42^

Civil servants from across DHSS were also anxious that their own divisions would be represented. Under Secretary Andrew Collier, from the Community Health Services Division, approached his colleagues and asked to be included.^43^ Admiral William Holgate, of the Dental Division, pointed out that there was no dentist on the steering committee and ended up getting the job himself.^44^ Dr H.G. Jones provided a link to the working party on collaboration with local government. The Permanent Secretary of the DHSS, Sir Philip Rogers, chaired the committee.

The steering committee oversaw a smaller ‘study group’, chaired by F.D.K. Williams, Assistant Secretary in the H3 Division, which did most of the detailed policy work. The apparent openness to external expertise and corporate ideas was demonstrated by the inclusion of Sir Richard Meyjes, head of marketing at Shell, who had been part of an influential group of business leaders appointed by Conservative Prime Minister Edward Heath to help improve civil service efficiency.^45^ Most importantly, however, the study group received expert advice from Brunel University’s Health Services Organization Research Unit, led by the influential Professor of Sociology Elliott Jaques, and management consultants from McKinsey & Co.

## 4 The McKinsey Moment?

US management consultants had followed leading US companies in expanding into Europe during the 1950s, selling American management ‘know-how’ to European companies anxious to learn how their new competitors were organised.^46^ The expansion of McKinsey was particularly important in Britain. They had considered and decided against a London office as early as 1953 and planned to work primarily for US subsidiaries, but this changed after increased demand from European companies on the back of prestigious work for high profile clients such as Shell and IBM. The London office was established in 1959 and by 1966 it was the group’s second largest.^47^ The multidivisional corporate structure was at the heart of their success. The firm was widely known in Britain by the late 1960s. Publicity accompanied the work done for famous British companies such as Dunlop, ICI, Cadbury, Cunard, Vickers, Tate and Lyle, Unilever and Rolls Royce. In the public sector British Rail, the BBC, the Bank of England and the Post Office also employed McKinsey for advice about restructuring.^48^

In the healthcare field, British consultancy firms had been advising individual hospitals about improving efficiency on a small scale since at least the early 1950s. The Ministry of Health had allowed a number of pro bono pilot studies in the NHS in 1958 and Urwick Orr were used to advise their architects on efficient systems for the Best Buy Hospital Scheme.^49^ At this stage, however, civil servants were cautious about the use of consultants, not wanting to undermine the autonomy of local hospitals or their responsibility for implementing their own efficiency measures. The use of management consultants was noteworthy but contained.

An important moment then came in 1968 when the board of Oxford United Hospitals invited McKinsey to look into their financial difficulties. The chairman, Eric Towler, head of the oil distributor Cawood’s, wanted to know whether they were underfunded or inefficient and badly managed. McKinsey concluded that it was ‘a bit of both’; there were grounds for thinking they were underfunded in relation to similar hospitals, and also that they were not very well managed.^50^ The proposed solution was to cut expenditure (one hospital was closed and its work absorbed elsewhere) and to strengthen management (a new organisational structure with a greater emphasis on clinical participation was designed).^51^ Small but impactful changes to hospital routines were introduced: starting the work of the x-ray department an hour earlier each day; customising the size of prescription dispensing containers to reduce waste. McKinsey’s reports also advised seeking opportunities for raising revenue, such as introducing car park charges. By considering questions of finance and management at a much broader level, the Oxford study was different in character and scope from those previously carried out by management consultants in the health care sector. NHS consultancy work represented a new direction for McKinsey. Special permission to undertake the study had to be sought from head office in New York.^52^ It was led by Robert Maxwell, who was developing an interest in the healthcare sector. He later worked as an administrator at St Thomas’ Hospital and was secretary and chief executive of the King’s Fund from 1980 to 1997. Though still only a small operation, the experience gained by the firm in Oxford was important.

Favourable publicity was secured when Maxwell wrote about the study in the *British Journal of Hospital Medicine* in 1969 and the story was picked up by the national press.^53^ Towler, along with the hospital administrator, John Spencer, and one of the consultant physicians, Peter Sleight, also produced an article for the *British Medical Journal*.^54^ They highlighted the advantages of using McKinsey – a team that had ‘time to study problems in depth’, and suggested that ‘the medical and administrative staff are far more likely to adopt a plan suggested by a disinterested outsider than one proposed from some internal group’.^55^ They described the McKinsey team as ‘three or four highly paid intelligent and sympathetic young men’ and praised their continuing interest in the Oxford NHS hospitals:

Their representatives continued to come to our committee meetings for many months after to make sure that our discussions were taking the right course, and that the changes they had recommended were being carried out, and that the administrative structure was running smoothly …They have done a difficult job intelligently and sympathetically. We hope this honeymoon period gives rise to a stable marriage.^56^

During the early 1970s Maxwell worked for McKinsey on a reorganisation of health services in the Republic of Ireland, a separation of public hospitals from the City administration in New York, and a study of psychiatric hospitals in the Netherlands.^57^ The firm first worked for the DHSS on an internal study of its own organisation.^58^ While social security was thought to be well administered, the health side was seen as ill prepared to manage the NHS after the planned reorganisation. The professional medical staff and non-professional civil servants had traditionally been kept separate with different responsibilities. Now they were brought closer together through joint working, and regional divisions and a central planning unit were introduced to strengthen national policymaking.^59^ Again, Maxwell led for McKinsey, liaising with the civil servant Ronald Matthews at DHSS.

In May 1971 McKinsey were subsequently invited to take part in the management study. They were chosen for their management expertise and because they were seen to add an ‘American flavour’.^60^ As discussed, consultancy firms founded in the US were increasingly successful in making their overseas advice appealing to British companies and public bodies during the 1960s. It was also recognised that there was insufficient internal support available. Even if it had been, civil servants judged that this ‘would not command public confidence in the same way as would the employment of consultants’.^61^ It was hoped that outside advice would be seen as independent and objective, and therefore more readily received if it touched upon complex and difficult questions.

Ultimately, however, the decision to go with McKinsey was underpinned by the fact that they were already known to the department:

Granted the case for employing outside consultants, the logic of using McKinsey’s seems to us indefeasible. First, there is the intrinsic value of the experience gained in their recent study of the Irish National Health Service and of hospitals in the USA we well as in our own NHS (in which they seem to us to have the edge on other leading consultants). Second there is the point that the management structure of the NHS must be complementary to that of the Department and this can most easily be achieved if the same consultants are working with us on both.^62^

While the work on the departmental study was put out for competitive tender, with McKinsey winning out over four other firms, that on the management study was not. Time was a key factor. Work on the required legislation was already underway and it was hoped that the management study would be completed inside eighteen months.^63^ A general view of how the process was likely to unfold was expected ahead of the publication of the White Paper planned for later in 1971. Permission had to be sought from the Civil Service Department (CSD) that monitored the use of consultants, but civil servants pushed hard for McKinsey to be used again.

The CSD were primarily concerned with expenditure. As such, they pressed for the study group to make the greatest possible use of civil servants and for the inclusion of the Brunel representatives, Jaques and Professor Maurice Kogan, who were seen as important participants. Their paper ‘The Future Structure of the National Health Service: Comments on the Second Green Paper’ was well known to civil servants. As John Archer of the CSD noted:

I know that he [Jacques] and Professor Kogan have staff working with them with experience of studies in the NHS and I have personally been impressed by the quality of some of Kogan’s writings on the subject of NHS organisation. It might both reduce the cost and bring fresh minds with rather different backgrounds to bear if there were some participation from this source at the study group level.^64^

The Brunel team were also thought to bring a degree of academic objectivity and had previous experience of working with complex organisations and the issue of ‘sapiential authority’.^65^

The individual consultants put forward by McKinsey were interviewed beforehand. Most were relatively junior, in their twenties or thirties. They included John Banham, who went on to be Director General of the Confederation of British Industry, and James Lee, who moved on to a career in the media, including as Chairman of the Bureau of Investigative Journalism. The Engagement Director overseeing McKinsey’s involvement – the partner responsible for all the work undertaken – was the American Henry Strage. Robert Maxwell concentrated on the internal study of the CSD, but also attended meetings of the study group on a regular basis. The first Engagement Manager for the project was Christopher Stewart-Smith, a future Chairman of the British Chambers of Commerce.

McKinsey initially quoted a figure of £11 000 per month for the services of three full time consultants and others when required on a part time basis, which was negotiated down to £10 000.^66^ The study was divided into three phases – ‘development of a feasible range of alternative organisational and process hypotheses’, ‘testing of hypotheses’, and ‘implementation’ – with McKinsey initially employed for Phase I, which would last six months. The motives of civil servants in this regard were interesting. As Archer of CSD noted, ‘There was, I admit, the presentational point made that if we have to answer a PQ [Parliamentary Question] about this new assignment, we could at this stage say that only £60 000 had been committed’.^67^ But he also confidently expected that McKinsey would not be needed for the full eighteen months of the study.

By December 1971 the firm’s fees had consistently come in at around £6000 a month, but this subsequently increased, and the slack in the overall budget was taken up once extra consultants were added during Phase II.^68^ Not everything was completed on time, but sufficient progress was made, so that a little after the expected end of Phase II in July 1972 the proposed management arrangements were agreed by the steering committee and put forward to Secretary of State Sir Keith Joseph. The result of the exercise was the famous ‘Grey Book’, *Management Arrangements for the Reorganised National Health Service*, which was published in September 1972.^69^

The Grey Book described in detail the functions of each new tier of the NHS and the duties and responsibilities of twenty-seven new roles. Members of the Regional Health Authorities (RHAs) were to be appointed by the Secretary of State. RHA staff included a medical officer, a nurse, a works officer, a treasurer and an administrator. Their main function was planning. Members of the AHAs were appointed by representatives of the RHAs, local authorities and members of non-medical and nursing staff. The Chairman was appointed by the Secretary of State. Their staff included a medical officer, a nurse, a treasurer and an administrator. They had planning and management functions and aimed to develop services with their corresponding local authority. Most Areas were split into health districts, with each District Management Team comprising an elected consultant, an elected GP, a community physician, a nurse, an administrator and a finance officer. They would manage and co-ordinate everyday services. An array of statutory and non-statutory professional advisory committees would also ensure professional involvement.^70^

The Grey Book also outlined the philosophy underpinning the new management arrangements at each level. Multidisciplinary teams would follow a process of ‘consensus management’. Each officer would be equal and decisions would be made collectively. If agreement could not be reached, then issues would be passed up the chain: ‘delegation downwards should be matched by accountability upwards’.^71^

Once the Grey Book had been published, DHSS civil servants were aware of the need to respond to the ‘strong and growing demand from the Service for leadership’.^72^ Phase III, which focused on implementation and communication, therefore included attempts to share progress and explain the reorganisation plans to doctors, nurses, dentists, administrators and professional groups across the country. Such a process had been foreshadowed in the White Paper, and a series of seminars and conferences were organised, with the aim of boosting morale. Six universities were signed up and expected to reach more than 2000 senior health officials.^73^ McKinsey played an important role in this ‘management education programme’, providing speakers, principally John Banham and James Lee, and materials for integration and training courses.^74^ This in particular may have helped to tie the McKinsey name to the reorganisation. Banham was also retained in a reduced role to advise on ongoing planning issues. He has subsequently suggested that NHS staff were about as well prepared as they could have been for the significant disruption that was coming.^75^

Aside from these expected roles, Strage tried unsuccessfully to get the full McKinsey team kept on for Stage III. His conception that the firm was needed in order to train DHSS officers to carry out the complex reforms was popular among some leading civil servants,^76^ but, as will be discussed below, others remained unconvinced and McKinsey’s role was slowly phased out within the eighteen month timeframe of the study.

## 5 The Reorganisation in Practice

The National Health Service Reorganisation Act reached the statute book in July 1973. ‘Shadow authorities’ were established to prepare for implementation and begin to make appointments, taking over from a series of local Joint Liaison Committees.^77^ The new structures came into effect on 1 April 1974. By then, Labour’s Barbara Castle had replaced Sir Keith Joseph as Secretary of State for Health and Social Services after a snap general election that February. She was more sceptical about the proposed changes than her predecessors, and within the first week of taking office had to decide whether to go ahead with the reforms. In a crucial meeting, the DHSS Permanent Secretary Sir Philip Rogers laid out the options. According to David Owen, newly appointed as Minister of Health:

He [Rogers] presented the case in considerable detail. The meeting must have been at least an hour and [he] very forcefully but pretty fairly presented this machinery that was being built up and everything like that, and then he ended and said, ‘Secretary of State, we are here to do what you want. It is possible to stop it. I have given you every form of argument why it would be very difficult but if you decide to do it we will loyally follow it through’.^78^

Castle decided that there was no alternative but to let the reorganisation continue. ‘Chaos’ would likely have ensued otherwise.^79^ Small changes were made to try and make the structures more ‘democratic’, including increasing the number of local authority representatives at the Area level.^80^ The option of removing the regional tier or making it advisory was discounted after it was discovered how far the implementation plans had already progressed. Thus, although there were differences between the two main political parties, they were in relation to the details of change not the overall principle, and they did not have a significant impact on the course of the reorganisation. The practical constraints that British health policymakers often find themselves working within are also clear. With regard to management consultants, it is likely that a Conservative government was instinctively more comfortable working with them, but there had long been a cross-party appreciation of the need for better management in the NHS during the early 1970s, and their role in the healthcare field was relatively confined and much less contentious than it would later become.

However, the reorganisation did not prove to be a durable solution to many of the problems that the NHS faced. The ‘dry rot of disillusionment’ soon appeared.^81^ A Royal Commission, chaired by Sir Alec Merrison, was established in 1976 to once again evaluate the long-term viability of the service. It heard evidence of increased bureaucracy, delays in taking difficult decisions and strained relationships between the administrative tiers.^82^ Conflict between the new FPCs and AHAs was common.^83^ In their evidence to the Commission even McKinsey admitted that the 1974 reorganisation had led to a ‘proliferation of paper’.^84^ In an evidence session in June 1978, John Banham summed up much that was thought to have gone wrong with the reforms:

With hindsight, it would have been better to have:

(a) avoided the creation of both area and district levels of management;

(b) had more discussion on whether there was a real role for the Regional tier;

(c) removed the requirement that areas should have the same boundaries as local government, and established a series of single-district areas;

(d) tested the reorganised management structure in, say, one region before adopting the plan nationally;

(e) had better selection arrangements for top jobs in the reorganised structure.^85^

Maxwell had also conceded that ‘It [was] very sad …that we did not do more trials of alternative forms of NHS reorganisation’, which would have provided ‘a better basis for the final form of the service’.^86^ Avoidable problems included ‘anxiety generated among staff of all disciplines by the handling of their translation from old to new appointments’ and ‘uncertainty in administrative units about their power and functions’. More widely he diagnosed a state of ‘anomie’ (‘spiritual impoverishment characterized by confusion and aimlessness’) in the NHS.^87^ It was, Maxwell argued, ‘in real peril’, partly as a result of ‘clumsy reorganisation’, but also due to other factors including the ‘militancy’ of NHS staff, ‘doctrinaire political attitudes’ and ‘chronic under-financing’.^88^ One potential answer was the introduction of non-medical chief executives in order to offset concerns about weak decision making, which had been tried in the Republic of Ireland. By the early 1970s such an idea had been around in health policy circles for some time, but it was rejected by the steering committee, as it had been at other times, as potentially representing an unacceptable challenge to medical authority.^89^ Once the 1974 reorganisation was complete, therefore, McKinsey representatives were willing to voice their frustrations, and in doing so provided an insight into the kinds of thinking, often quite dramatic thinking, that had not taken hold, or not been allowed to emerge at all during the management study. Maxwell himself, outside of project constraints, was engaged in much broader thinking about the challenges facing the NHS and ensuring it had a viable future, including difficult issues such as health care rationing and levels of public spending.^90^

Following the publication of the Royal Commission’s report and a return to Conservative government in May 1979, a series of further reorganisations took place: AHAs were abolished in 1982; consensus management was replaced by ‘general management’ after the 1983 Griffiths NHS Management Enquiry; an NHS Management Board was created in 1985 and an NHS Executive in 1989; the internal market was introduced in 1991. Further local and regional reconfigurations followed regularly throughout the 1990s and 2000s, and the most recent, the 2012 Health and Social Care Act, created Clinical Commissioning Groups. Little evidence of those first significant changes in 1974 remains.

## 6 ‘We Have Got a Problem’

One of the most intriguing aspects of the 1974 reorganisation is the extent to which many of those involved could see problems emerging soon after its completion, or even while it was still being planned. It was only natural that in an organisation as large and complex as the NHS the reorganisation would not be completely successful, but many of its weaknesses were built in from the start.

Timing was a key issue. When reflecting on the management study in 1975 the DHSS civil servant F.D.K. Williams was already clear that:

Ideally the study should have been undertaken at an earlier stage of planning NHS reorganisation. This was not possible for political reasons. Accordingly the framework for reorganisation was decided before the study was commissioned. The consultants showed themselves able to work within this framework, though it was less than ideal.^91^

In this respect, we can see reflections of ongoing concerns about health policymaking being subject to a political timetable that does not align with the interests of the service itself. Reform appeared feasible because there was a sufficient measure of agreement between the two main political parties and key interest groups. There was a sense of inevitability behind the reforms; they were part of an evolutionary process. But in the short term they were still subject to unavoidable political and legislative calculations. Health reform is routinely on the political agenda, because of the enduring imperfection of the NHS and perpetual uncertainty about its future. There may not be a perfect time for change in the NHS, but restrictive deadlines can particularly hamper successful policymaking. The infamous ‘pause’ of the coalition government’s NHS reforms in 2010 also points to this.

The outcome of the 1974 NHS reorganisation speaks to the continuing power of the medical profession. It was a compromise. As long as doctors’ opposition to more radical changes remained strong there was no serious possibility of considering options such as moving all health services under local government control or introducing non-medical chief executives. At the beginning of the management study Joseph had made clear his ‘intention of constructing the strongest management structure consistent with …the preservation of clinical freedom’.^92^ In the event, the strength of the management structure was debatable. With the advent of consensus management, medical professionals may even have ended up with more of a voice in the running of the service than they had before.^93^

The issue of accountability and where it truly lay – another perennial theme in NHS history – was not adequately addressed during the 1974 reorganisation. As Klein observes, ‘The rhetoric was all about accountability and monitoring. But what was the currency of accountability and where were the tools of monitoring?’^94^ These are difficult concepts to pin down, particularly in a large and complex organisation like the NHS, but on the ground, in the hospitals and clinics, the reorganisation created uncertainty. Although doctors were clinically accountable to their Executive Council and the General Medical Council, it was not made clear who was ultimately responsible for the successful running of services. Indeed, no one figure was responsible. This ‘problem’ was quickly returned to by the NHS Management Inquiry in 1983, exemplified by Roy Griffith’s famous observation that ‘if Florence Nightingale were carrying her lamp through the corridors of the NHS today she would almost certainly be searching for the people in charge’.^95^ But in 1974, discussion of accountability was ‘relatively meaningless’. As DHSS civil servant Eric Caines has noted, ‘When everyone is responsible no one is responsible’.^96^ Questions of who is ultimately responsible for NHS outcomes, and particularly who is being held accountable for the successful planning and implementation of wide ranging reforms, have been asked again during recent attempts at NHS reform.^97^

Nonetheless, it is important to recognise that the consensus management compromise arrived at in 1974 was actually seen as a positive in areas that were well suited to it. For example, Bob Nicholls, then District Administrator in Southampton and South West Hampshire, and later an influential Regional General Manager, recalls that ‘overall consensus management worked very well for us’.^98^ Questions of theoretical accountability mattered less when issues could be resolved. However, as before, this was often a result of local circumstances and the work of strong leaders on the ground. In the majority of places that were not prepared and had not already been doing something similar on an informal basis, consensus management could be a ‘shock’ or even a ‘disaster’.^99^

Another significant, but under-appreciated rationale for the reorganisation was that it would simply help to save money. Financial pressures have been a recurrent theme throughout the history of the NHS. Sir Keith Joseph was interested in modernisation, but he was also aware of the bottom line. Caines recalls: ‘He was handling a big budget on Social Security and an insufficient budget on Health and it was a time of financial crisis. There was a real practical edge to his desire to get this thing moving.’^100^ Although the primary aim was integration, if the financial pressures on the NHS could also be eased in the medium term, all the better.

The 1974 reorganisation also illuminates a number of important points about the use of management consultants in the health sphere. The involvement of McKinsey consultants in the management study speaks directly to current questions about why consultants are hired by health policymakers, the quality of their work, their specialist knowledge of and concern for health policy, the relative lack of expertise inside the NHS and the civil service, the potential lack of accountability and responsibility attached to consultancy work, its cost and wider pressures on resources, and the ways in which it can be used to add legitimacy to decision-making processes.

From 1968 CSD had kept track of work done by consultants for government departments. In March 1972, Management Services Branch 1, responsible for this monitoring in DHSS, asked for a ‘frank assessment of the value’ of McKinsey’s involvement in the management study.^101^ Caines argued that this would be ‘inappropriate’ while the study was still ongoing.^102^ A second attempt in February 1973 saw Williams reflect on McKinsey’s flexibility and methodical approach.^103^ When a further request came through in February 1974, Williams, who had since moved on inside DHSS, was more candid:

With two exceptions the consultants work tended to be superficial. This was hardly surprising considering the complexity and the extent of the field they had to deal with in a comparatively short time.^104^

By the time McKinsey were invited to take part in the management study in May 1971, many of the most important questions had already been closed off. Christopher Stewart-Smith quickly came to wonder why Sir Keith Joseph had hired McKinsey to implement decisions that had already been taken. When undertaking new work for a client, McKinsey were used to studying an issue, making an assessment and coming up with recommendations, before usually being asked to implement them. Here, the new administrative structure of the NHS had already been settled and was outside the remit of the study group. Banham and others were able to look back later and see that the arrangements were flawed, but it had already been apparent to some observers at the time. Even if Stewart-Smith and others suspected that the structures might not work, they could do little about it. Some observers were even clear that a further significant reorganisation was inevitable at some point.^105^ The failure to undertake pilot studies before committing to such significant reforms has come to be seen as another shortcoming, one which has been repeated in the course of subsequent NHS reforms. Stewart-Smith recalls:

I went to Sir Keith Joseph and said, ‘Look, we have got a problem with these two aspects and in particular the cost of the structure and the fact we are not piloting anything’. And he [Joseph] said, ‘I have decided what we are going to do’, and he gave me all the reasons why he believed in management and planning and all these things that people believed in at that time, and he said that if we did not want to do the job, basically he would find somebody else to do it. I took the view that McKinsey were very good at doing this sort of thing so they could do it but I was not going to be part of it.^106^

As a result, Stewart-Smith stepped down from the project and was replaced as Engagement Manager by Roderick Taylor. Although some ‘trials’ of the study group’s ‘tentative hypotheses’ were held in a number of areas, a wider pilot study of the reorganisation was not attempted.^107^ Concerns about the direction of the policymaking process had also been raised by figures like Meyjes, who resigned from the steering committee and the study group in March 1972 once it became clear that more ambitious proposals for reform based around greater autonomy for a central director general and regional chief executives would not be taken up, and the organisational path established by Crossman would be followed.^108^

In addition, the wider management solution that McKinsey might have been instinctively most comfortable proposing, based on their experience at Oxford in 1968, did not fit with the direction the study was likely to take. The 1971 McKinsey team had to compromise with civil servants, administrators and medical professionals – the other members of the study group and the steering committee – and work within the parameters that they set. There was no scope to revisit the recommendations of the consultative document on the structure of the NHS. Williams later reflected positively that ‘They [McKinsey] were quick to appreciate what were the practical possibilities taking account of the attitudes of the various health service professions and also flexible enough to amend their original organisational hypothesis quite radically’.^109^ Compromise was one of the defining features of the reorganisation as a whole. A decision-making process, designed by civil servants to have so many different voices feeding into it, was always likely to find a middle way; consensus management was the result. According to Caines, civil servants effectively controlled the process and steered it to a satisfactory, at least in the short term, conclusion:

We were prepared with catchphrases: consensus management, delegation downwards, accountability upwards. They all tripped off the tongue nicely. They accorded with the flavour of what the Steering Committee felt about how it should be run …When we went there we knew what we wanted and we knew what we were giving them. It went relatively smoothly as far as I am concerned.^110^

## 7 Value for Money

Securing value for money when using consultants was a key concern. This is clear from a debate in 1972 about whether McKinsey should be kept on for Stage III of the management study. At the beginning of the study CSD’s Archer had advised his DHSS colleagues that ‘the more senior McKinsey people represent value for their high fees but the more junior consultants, often just out of business school, do not’.^111^ The per diem rate for Strage, as Engagement Director, was £229. For his most junior colleague it was £104. Similar trade-offs between experience and cost continue today. Although the project ran to budget, the sceptical civil servant S. Bayfield, who had been part of the study group, pointed out that the cost of one McKinsey representative was the same as three Under Secretaries: ‘Viewed impartially and looking at the calibre of the personnel on the team this is very poor value for money.’^112^

Having invested in McKinsey at Stage I and II, it was a point of contention whether it made financial sense to continue with them for Stage III. Williams was among those who thought they should be brought back:

I am sure that if we discontinued the assignment at the end of July we should not yet have got optimum value out of our investment. In any case we need to get a rather larger nucleus of DHSS officers than at present familiar with the study and its concepts in order to carry the Study through without loss of impetus once the McKinsey team…have left.^113^

Bayfield was less sure:

As a general rule it is advisable to use consultants for the shortest possible time to the maximum possible effect and they are particularly strong on conceptual proposals but I suspect very weak on applications because of their total lack of experience in background.^114^

Considerable thought was also given to whether sufficient knowledge and manpower was available within DHSS. John Orme argued that ‘McKinseys have done and are doing well; and that we shall be the losers if we cut away from them’.^115^ Bayfield and others remained unconvinced, recognising the logic of bringing McKinsey back, but suggesting that a team of DHSS or NHS officers would be better placed to undertake the work needed in the next phase. Bayfield noted: ‘They are constantly exposing the inadequacy of their background and experience and to involve them [in Stage III] is in my view misconceived.’^116^

The issue of how close the relationship between external consultants and health policymakers should become was also recognised in 1972 by Assistant Under Secretary of State in DHSS, and later Permanent Secretary, Kenneth Stowe: ‘McKinsey’s have now had three successive major assignments and it is undesirable, as a matter of good practice, to develop a semi-permanent relationship with one firm.’^117^ There were also contemporary concerns about the impact on service efficiency. As Bayfield cautioned:

If the provision of manpower can only be obtained by using McKinseys, then we must accept the disadvantages of using inexperienced people for practical applications which require substantial experience. In the long run this may turn out to be more damaging to the efficiency of the service than one would wish.^118^

With the balance among civil servants against retaining McKinsey in full for Phase III but continuing concerns about the impact on the timetable for reorganisation planning, it was decided to try and equip civil servants with the right knowledge and experience and wind down the firm’s involvement, phasing it out by the end of 1972. The final McKinsey work, with only Banham and Lee retained in reduced roles, was seen as an extension of Phase II rather than a move to Phase III.

In the end, influenced by the wider economic crises of the 1970s, perhaps even subconsciously, McKinsey did not actively seek further consultancy work in the British health sector for a time after 1973.^119^ They later returned strongly, alongside their peers, during the 1980s and 1990s as part of a significant expansion in consultancy activity underpinned by wider managerial and governance related changes – a period described by Klein as a ‘gold rush’.^120^ Important opportunities were provided by the rolling out of NHS Trust status during the early 1990s, the subsequent development of ‘turnaround teams’ brought in to help Trusts that found themselves in financial difficulty during the early 2000s, and the development of NHS IT systems overseen by Department of Health agencies like ‘Connecting for Health’. From relatively humble beginnings, therefore, management consultants have steadily become culturally embedded in British health policymaking.

## 8 Conclusion

The 1974 reorganisation represents an important moment in the ongoing story of management consultants engaging with British health policymakers. It marked the first time that consultants had been engaged on a project of such importance in the healthcare field. They now play a significant role throughout the NHS and across Whitehall. In 2013–14 NHS spending on management consultants reached a peak of £640 million. Though this figure has since fallen back, to £371 million in 2017–18, and represents a relatively small component of the total NHS budget of around £125 billion, the underlying implications are significant.^121^ Many debates about the use of external consultants today were already apparent during McKinsey’s involvement in the 1974 NHS reorganisation. By the early 1970s consultants had been engaging with health policymakers for some time. McKinsey were a high profile firm and their healthcare experience, though not yet extensive, was well known. Their engagement in the management study was significant. It speaks to the emerging role of consultants in health policymaking, particularly as a response to a perceived lack of specialist knowledge or sufficient manpower inside the civil service and the NHS.

Nonetheless, although their contribution to the reorganisation was important in a number of respects, McKinsey were only one of the expert groups feeding into the process. The management study was structured so that different voices would be heard. This constrained the McKinsey representatives and steered the participants towards a compromise. Although the firm has come to be closely associated with the reorganisation and its failure, the analysis presented here suggests that this not need necessarily have been the case. For example, in 1975 a critique noted that ‘Management experts who understand little medical care were given free rein’.^122^ This was not strictly true, but it chimed with some medical practitioners’ beliefs. Since then it has become convenient shorthand, particularly in the wake of subsequent controversial reforms, to link 1974 to the McKinsey brand. This article has not sought to challenge the established historical assessment of the 1974 NHS reorganisation as an unsuccessful exercise. Rather, the objective has been to focus on management consultants and better understand the process by which it was brought about, the wider context and powerful institutional and professional cultures, and the roles played by different policy actors. Archival sources and the discussions at the witness seminar held in November 2016 have created a richer picture and allow for a nuanced assessment of the reforms.

The 1974 reorganisation was a product of its time, particularly the desire for modernisation and rationalisation in the planning of public services. However, its defining characteristics were ultimately pragmatism and compromise. This was likely unavoidable. Civil servants and government ministers were acutely aware of the need to accommodate key interest groups, not least those inside the NHS who would have to make the new arrangements work, particularly the medical profession. As Klein describes, ‘The search for an organisational solution to the NHS’s problems can therefore be best understood as policymaking under constraints, where the ideal was often seen as the enemy of the feasible: the politics of the second-best’.^123^ We can only speculate whether a more hard-headed and divisive approach that attempted to push through a more integrated service would have been more successful and long lasting. In the event, the NHS Gordian knot remained uncut.

Some of the flaws in the tripartite structure that had been in place since 1948 were addressed, and it is likely that the better organisation and co-ordination that resulted ultimately led to improvements in patient care. As discussed, by the late 1960s the pressure for reform had built such that it could not be put off any longer. For some NHS staff, particularly those who were well prepared and amenable, the reorganisation was not disruptive, and may even have worked well, but the new structures and processes did not suit everyone. They also produced new layers of bureaucracy and introduced new problems that had to be overcome. Full integration, which many academic observers believed was necessary, was not achieved; indeed it was not aimed for. In effect policymakers were attempting ‘to make substance from shadows’.^124^

Nonetheless, many of those who were directly involved in the reorganisation, whether through the study group and the steering committee of the management study, or on the ground in the NHS, saw problems ahead before the reforms even took effect. There was recognition that the expected cost savings were unlikely to be realised or that the required managerial efficiency would be generated. Many of the ‘mistakes’ that were made – over-prescription and direction from the centre, unnecessary bureaucracy, a lack of certainty about where ultimate responsibility lay, inadequate piloting – now appear explicable, if not excusable. Ultimately, however, this speaks to the poor institutional memory of Whitehall, and the Department of Health and Social Care in particular. When significant changes are not well planned and well implemented there is negative impact on resources, staff morale and service delivery.

The 1974 NHS reorganisation demonstrates the extent to which British health policymakers have had to work within cultural, political, legislative and practical constraints when trying to initiate change. In 1974 McKinsey were not yet performing the wide ranging and controversial role in the health sphere that management consultants would take on in future, particularly from the 1980s and 1990s as managerial and market-oriented reforms were introduced and a normative culture that facilitated a significant role for external consultants developed. But their involvement in the 1974 reforms speaks directly to ongoing questions about the ways in which consultants come to be hired by health policymakers (often through reputation or established relationships), the quality of their work, the perceived lack of expertise inside the civil service and the NHS, and the ways in which they appear to legitimise decision-making processes. McKinsey were seen as managerial experts in the early 1970s. Ultimately, however, the 1974 NHS reorganisation was defined by practical rather than philosophical considerations, and external management experts could make only a limited contribution to an imperfect compromise.

## Footnotes

1.
N. Timmins, *Never Again? The Story of the Health and Social Care Act 2012, A Study in Coalition Government and Policy Making* (London: Institute for Government, 2012), 13.

2.

M. Boussebaa , A. Sturdy and G. Morgan , ‘Learning from the World? Horizontal Knowledge Flows and Geopolitics in International Consulting Firms’, International Journal of Human Resource Management, 25, 4 (2014), 1227–1242.

3.

C. McKenna , The World’s Newest Profession: Management Consulting in the Twentieth Century (Cambridge: Cambridge University Press, 2006).

4.
S. Knapton, ‘NHS Spending on Management Consultants Doubles Under Coalition’, *Daily Telegraph*, 9 December 2014.

5.
S. Neville, ‘Health Consultancy Work Under Pressure as NHS Seeks Savings’, *Financial Times*, 18 January 2018; C. Ham, H. Alderwick, P. Dunn and H. McKenna, *Delivering Sustainability and Transformation Plans: From Ambitious Proposals to Credible Plans* (London: King’s Fund, 2017).

6.
K. Clarke, in E. MacKillop, S. Sheard, P. Begley and M. Lambert (eds), *The NHS Internal Market* (University of Liverpool, 2018), 19. Transcript available at:https://www.liverpool.ac.uk/media/livacuk/iphs/researchgroups/governanceofhealth/Internal,Market,WS,transcript,ESM,15.06.2018.pdf.

7.
C. Webster, *The Health Services Since the War, Volume II. Government and Healthcare: The National Health Service 1958–79* (London: The Stationery Office, 1996); G. Rivett, *From Cradle to Grave: Fifty Years of the NHS* (London: King’s Fund, 1998); R. Klein, *The New Politics of the NHS: From Creation to Reinvention* (London: CRC Press, 2013).

8.

National Health Service Reorganisation Act(London: HMSO, 1973).

9.
R. Levitt, *The Reorganised National Health Service* (London: Croom Helm, 1976); R.G.S. Brown, *Reorganising the National Health Service* (Oxford: Basil Blackwell, 1979).

10.
Levitt, *ibid.*, 208.

11.
Brown, *op. cit.* (note 9), 164.

12.
J. Lewis, *What Price Community Medicine? The Philosophy, Practice and Politics of Public Health Since 1919* (Brighton: Wheatsheaf, 1986); S. Harrison, *Managing the National Health Service: Shifting the Frontier?* (London: Chapman and Hall, 1988).

13.
P. Begley, E. MacKillop and S. Sheard (eds), *The 1974 NHS Reorganisation* (University of Liverpool, 2017). Transcript available at: https://www.liverpool.ac.uk/media/livacuk/iphs/researchgroups/governanceofhealth/PDF1,-,1974_.pdf.

14.
C. Webster, *National Health Service Reorganisation: Learning From History* (London: Office of Health Economics, 1998), 13; R. Klein, *op. cit.* (note 7), 71.

15.
G. O’Hara, *From Dreams to Disillusionment: Economic and Social Planning in the 1960s* (Basingstoke: Palgrave Macmillan, 2006); R. Lowe, *The Welfare State in Britain Since 1945* (Basingstoke: Macmillan, 1993).

16.
Webster, *op. cit.* (note 14), 8.

17.
While the provenance of the original quote is unclear, the best account is that outlined in S. Sheard, ‘A Creature of its Time: The Critical History of the Creation of the British NHS’, *Michael Quarterly*, 8, 4 (2011), 428–41.

18.
Brown, *op. cit.* (note 9), 8.

19.
Webster, *op. cit.* (note 14), 12.

20.

Report of the Committee of Enquiry into the Cost of the National Health Service(London: HMSO, 1956).

21.

*A Review of the Medical Services in Great Britain* (London, Social Assay, 1962).

22.

*Ibid.*, 158.

23.
Begley *et al.*, *op. cit.* (note 13), 16.

24.

*Ibid.*, 19.

25.
Brown, *op. cit.* (note 9), 10.

26.
Hansard, HC Deb 08 August 1966, Vol. 733, Col.1151.

27.

M. Gorsky , ‘The British National Health Service 1948–2008: A Review of The Historiography’, Social History of Medicine, 21, 3 (2008), 444.

28.

*Report of the Committee on Senior Nursing Staff Structure* (London: HMSO, 1966); *First Report of the Joint Working Party on the Organisation of Medical Work in Hospitals* (London: HMSO, 1967).

29.

*Royal Commission on Local Government in England*, Cmnd. 4040 (London: HMSO 1969).

30.

The Administrative Structure of the Medical and Related Services in England and Wales(London: HMSO, 1968).

31.
Klein, *op. cit.* (note 7), 68.

32.
Begley *et al.*, *op. cit.* (note 13), 20.

33.

The Future Structure of the National Health Service(London: HMSO, 1970).

34.

R.M. Battistella and T.E. Chester , ‘The 1974 Reorganization of the British National Health Service: Aims and Issues’, The New England Journal of Medicine, 289, 12 (1973), 610–15.10.1056/NEJM1973092028912054198820

35.

National Health Service Reorganisation: Consultative Document(London: HMSO, 1971).5206987

36.
Klein, *op. cit.* (note 7), 69.

37.

National Health Service Reorganisation: England(London: HMSO, 1972).

38.
Brown, *op. cit.* (note 9), 48.

39.
The National Archives, MH/159/377, ‘NHS Reorganisation: Study of Internal Organisation of Health Authorities’, 1 April 1971.

40.

*Ibid.*, Cashman to Dodds, 25 March 1971.

41.

*Ibid.*, Mayston to Bavin, 12 May 1971.

42.

*Ibid.*, Hughes to Mason, 17 May 1971.

43.

*Ibid.*, Collier to Orme, 19 April 1971.

44.

*Ibid.*, Holgate to Orme, 10 May 1971.

45.

K. Theakston , ‘The Heath Government, Whitehall and the Civil Service’, in S. Ball and A. Seldon (eds), The Heath Government 1970–74: A Reappraisal (London: Longmans, 1996), 80.

46.
McKenna, *op. cit.* (note 3).

47.

*Ibid*.

48.

*Ibid*.

49.
Interview with Tim Nodder, October 2016.

50.
R. Maxwell, ‘Health Policy, Management and Gardening’ in A. Oliver, *Personal Histories in Health Research* (London, Nuffield Trust, 2005), 207–20.

51.
Interview with Robert Maxwell, May 2016.

52.
Interview with Henry Strage, June 2016.

53.

R. Maxwell , ‘How Sick are the Hospitals?’, British Journal Hospital Medicine (April 1969), 895–899.

54.

P. Sleight , J.A. Spencer and E.W. Towler , ‘Oxford and McKinsey: Cogwheel and Beyond’, British Medical Journal (14 March 1970), 682–684.10.1136/bmj.1.5697.682PMC17006015443973

55.

*Ibid*., 684.

56.

*Ibid*.

57.

*Op. cit.*, (note 50); *op. cit.*, (note 51).

58.
See Levitt, *op. cit.* (note 9), 32.

59.
Begley *et al.*, *op. cit.* (note 13), 32.

60.

*Ibid.*, 34.

61.
TNA, MH 159/383, Stowe to Archer, 23 April 1971.

62.

*Ibid*.

63.
TNA, MH 159/383, Orme to Parker, 6 May 1971.

64.

*Ibid.*, Archer to Stowe, 29 April 1971.

65.
Begley *et al.*, *op. cit.* (note 13), 33. Sapiential authority refers to the right to be heard based on knowledge and experience rather than structure or position.

66.
TNA, MH 159/383, Strage to J.S. Orme, 11 June 1971; Strage to Orme, 6 July 1971.

67.

*Ibid.*, Archer to Orme, 8 July 1971.

68.

*Ibid.*, Strage to Williams, 11 January 1972.

69.

Management Arrangements for the Reorganised National Health Service(London: HMSO, 1972).

70.
Brown, *op. cit.* (note 9), 125.

71.

*Op. cit.* (note 69), 10.

72.
TNA, MH 159/383, Hulme to Dodds, 19 April 1972.

73.
Battistella and Chester, *op. cit.* (note 34), 615.

74.
TNA, MH 159/383, Strage to Williams, 30 October 1972; Strage to Williams, 16 January 1973; Strage to Williams, 18 April 1973.

75.
Interview with Sir John Banham, August 2016.

76.
TNA, MH 159/383, Strage to Williams, 19 May 1972.

77.
Brown, *op. cit.* (note 9), 50-3.

78.
Begley *et al.*, *op. cit.* (note 13), 42.

79.

*Ibid*.

80.

Democracy in the National Health Service: Membership of Health Authorities(London: HMSO, 1974).

81.
Klein, *op. cit.* (note 7), 90.

82.

*Report of the Royal Commission on the National Health Service* (London: HMSO, 1979), Cmnd. 7615.

83.

S. Ainsworth , ‘A Rose-Tinted Spectacle’, Health Service Journal (1 April 1999), 20–21.10387794

84.

M. Walker , ‘NHS Should Charge Patients, say McKinsey Consultants’, The Guardian (24 June 1977).

85.
TNA, BS 6/3511 ‘Session with John Banham, McKinsey & Co.’.

86.

R. Maxwell , ‘Anomie in the NHS: A McKinsey View’, British Medical Journal (16 August 1975), 424.PMC16738801156796

87.

*Ibid*.

88.

*Ibid*.

89.

D.J. Hunter , ‘Consensus Management of Chief Executives? Lessons from the NHS’, Local Government Studies, 10, 3 (1984), 39–50.

90.

*Op. cit.* (note 86), 425.

91.
TNA, MH 159/383, Williams to Owen, 7 February 1974.

92.

*Ibid.*, Stowe to Archer, 28 April 1971.

93.
Klein, *op. cit.* (note 7), 69.

94.

*Ibid*., 92.

95.

M. Gorsky , ‘“Searching for the People in Charge”: Appraising the 1983 Griffiths NHS Management Inquiry’, Medical History, 51, 1 (2013), 89.10.1017/mdh.2012.82PMC356675323393404

96.
Begley *et al.*, *op. cit.* (note 13), 34.

97.

J. Hammond , C. Lorne , A. Coleman , P. Allen , N. Mays , R. Dam , T. Mason and K. Checkland , ‘The Spatial Politics of Place and Health Policy: Exploring Sustainability and Transformation Plans in the English NHS’, Social Science and Medicine, 190 (2017), 217–226.2886647510.1016/j.socscimed.2017.08.007

98.
Begley *et al.*, *op. cit.* (note 13), 44.

99.

*Ibid*.

100.

*Ibid.*, 20.

101.
TNA, MH 159/383, ‘Use of Management Consultants By Government Departments’, 29 March 1972.

102.

*Ibid.*, Caines to Marsh, 19 April 1972.

103.

*Ibid.*, Williams to Marsh, 2 March 1973.

104.

*Ibid.*, Williams to Owen, 7 February 1974.

105.
Battistella and Chester, *op. cit.* (note 34), 614.

106.
Begley *et al.*, *op. cit.* (note 13), 21.

107.
Brown, *op. cit.* (note 9), 48.

108.

C. Webster , The National Health Service: A Political History (Oxford: Oxford University Press, 2002), 102.

109.
TNA, MH 159/383, Williams to Owen, 7 February 1974.

110.
Begley *et al.*, *op. cit.* (note 13), 35.

111.
TNA, MH 159/383, Archer to Orme, 11 May 1971.

112.

*Ibid.*, Bayfield to Bourton, 25 May 1972.

113.

*Ibid.*, Williams to Orme, 23 May 1972.

114.

*Ibid.*, Bayfield to Bourton, 25 May 1972.

115.

*Ibid.*, Orme to Dodds, 30 May 1972.

116.

*Ibid.*, Bayfield to Bourton, 25 May 1972.

117.

*Ibid.*, Stowe to Gedling, 30 June 1972.

118.

*Ibid.*, Bayfield to Bourton, 7 June 1972.

119.

*Op. cit.* (note 51).

120.
Klein, *op. cit.* (note 7), 166.

121.
Department of Health and Social Care, *Annual Report and Accounts 2017–18* (London: DHSC, 2018); D. Oliver, ‘Awkward Questions about NHS Management Consultancy’, *British Medical Journal*, 10 December 2016, 442–3.

122.

S. Jonas and D. Banta , ‘The 1974 Reorganization of the British National Health Service: An Analysis’, Journal of Community Health, 1, 2 (1975), 103.10.1007/BF01319203819465

123.
Klein, *op. cit.* (note 7), 67.

124.
Jonas and Banta, *op. cit.* (note 122), 104.

